# Power Control in Wireless Body Area Networks: A Review of Mechanisms, Challenges, and Future Directions

**DOI:** 10.3390/s26030765

**Published:** 2026-01-23

**Authors:** Haoru Su, Zhiyi Zhao, Boxuan Gu, Shaofu Lin

**Affiliations:** 1 College of Computer Science, Beijing University of Technology, Beijing 100124, China; suhaoru@bjut.edu.cn (H.S.); 15726951774@163.com (Z.Z.); 2 Beijing-Dublin International College, Beijing University of Technology, Beijing 100124, China; guboxuan@emails.bjut.edu.cn

**Keywords:** wireless body area network (WBAN), power control, energy efficiency

## Abstract

Wireless Body Area Networks (WBANs) enable real-time data collection for medical monitoring, sports tracking, and environmental sensing, driven by Internet of Things advancements. Their layered architecture supports efficient sensing, aggregation, and analysis, but energy constraints from transmission (over 60% of consumption), idle listening, and dynamic conditions like body motion hinder adoption. Challenges include minimizing energy waste while ensuring data reliability, Quality of Service (QoS), and adaptation to channel variations, alongside algorithm complexity and privacy concerns. This paper reviews recent power control mechanisms in WBANs, encompassing feedback control, dynamic and convex optimization, graph theory-based path optimization, game theory, reinforcement learning, deep reinforcement learning, hybrid frameworks, and emerging architectures such as federated learning and cell-free massive MIMO, adopting a systematic review approach with a focus on healthcare and IoT application scenarios. Achieving energy savings ranging from 6% (simple feedback control) to 50% (hybrid frameworks with emerging architectures), depending on method complexity and application scenario, with prolonged network lifetime and improved reliability while preserving QoS requirements in healthcare and IoT applications.

## 1. Introduction

Wireless Body Area Networks (WBANs), defined as low-power, short-range wireless sensor networks operating in close proximity to, on, or inside the human body for medical and non-medical applications [1,2], enable real-time data collection for medical monitoring, sports tracking, and environmental sensing within the healthcare Internet of Things (IoT) [2]. For medical-grade WBAN devices, compliance with regulatory frameworks such as the FDA (United States) and CE marking (European Union) is mandatory to ensure clinical safety, data integrity, and therapeutic efficacy. Their low-power, short-range design supports wearable and implantable devices for applications like remote healthcare and fitness tracking [2]. However, limited battery life and dynamic environments—particularly body motion and channel fading—pose significant challenges to energy efficiency [3,4]. Body motion directly impacts WBAN performance by inducing dynamic channel fading and shadowing: limb movements alter signal propagation paths, causing received signal strength to fluctuate substantially and increasing bit error rates, which necessitates adaptive power control to maintain reliability during activities like walking or exercising [3,4].

**Figure 1:**
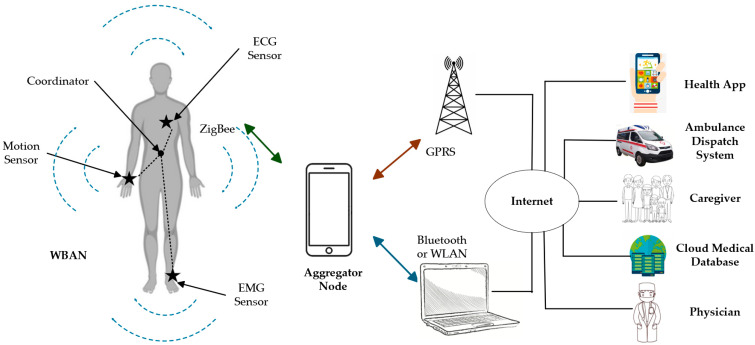
System architecture of Wireless Body Area Networks.

To address these, WBANs employ a layered architecture that facilitates efficient data collection, transmission, and processing while optimizing energy allocation across components [1,2]. Sensor nodes are low-power sensors that collect physiological data, such as heart rate or blood oxygen, or environmental data, like temperature or humidity, with limited computational capacity, designed for wearability or implantation [2,5]. Sink nodes aggregate data, perform compression, and forward it via Bluetooth or ZigBee to external devices, equipped with higher computational and storage capabilities (as illustrated in Figure 1) [1]. Remote servers analyze and store data, leveraging machine learning for predictive analytics, such as disease detection or activity recognition [4]. Application layers provide user interfaces for real-time monitoring and feedback, enabling applications like health alerts or performance tracking [2].

Energy consumption in WBANs arises from several key sources critical to network performance [3,5]. Transmission dominates as the primary energy drain, depending on distance, power levels, and data rate [3]. Idle listening, where nodes monitor channels during low activity, leads to energy waste [5]. Collision retransmissions, caused by interference in multi-node settings, further increase energy costs [3]. Data processing and hardware, including sensing, compression, and static leakage in radio frequency circuits, also contribute to consumption [5]. Notably, transmission accounts for over 60% of total energy use, making it essential to address these sources for targeted power control strategies that minimize waste and extend network lifetime in energy-constrained applications like medical monitoring [3,5]. Power control dynamically adjusts node transmission power to optimize energy use while ensuring QoS requirements, thereby extending network lifetime for sustainable healthcare IoT applications [3,4].

Existing surveys on WBANs, such as [2], provide broad overviews of architectures and applications, while [3] offers a taxonomy of transmission power control (TPC) mechanisms based on link quality estimators and power level control strategies. Recent works like [4] introduce lightweight deep learning for channel prediction, achieving long-term accuracy in non-stationary channels, and [5] explores energy harvesting for self-powered sensors through body heat, motion, or ambient sources. However, these often lack deep integration of AI-driven prediction with energy harvesting or standardized comparisons in dense deployments. This paper reviews power control mechanisms for WBANs, focusing on energy efficiency and healthcare IoT applications, addressing these gaps through a comprehensive analysis. Beyond algorithmic and architectural considerations, the practical deployment and regulatory acceptance of WBANs are fundamentally shaped by communication standards and safety regulations. The IEEE 802.15.6 standard [6]—explicitly designed for short-range, low-power wireless communication in and around the human body—provides the physical (PHY) and medium access control (MAC) layer foundation, supporting multiple PHY options (narrowband, ultra-wideband, and human body communication) with built-in quality-of-service (QoS) prioritization and energy-aware operation. Compliance with this standard, along with regional medical device regulations such as the FDA (U.S.) and CE marking (EU), imposes strict requirements on transmission reliability, frequency bands, interference limits, and patient safety. Consequently, any power control strategy discussed in this review must ultimately operate within these standardized and regulatory boundaries to ensure clinical viability and safe integration into healthcare IoT systems.

Our contributions include:

1. A systematic classification of TPC strategies (e.g., feedback, optimization, RL/DRL, hybrids) with quantitative insights into energy savings;
2. Integration of emerging techniques like energy harvesting and AI-driven prediction;
3. Insights into challenges like algorithmic complexity and privacy, with future directions for scalable deployments.

The remainder of this paper is organized as follows: Section 2 describes the review methodology, Section 3 surveys various power control mechanisms, Section 4 discusses key challenges and future research directions, and Section 5 concludes the paper.

## 2. Review Methodology

This paper presents a narrative review of recent advances in power control mechanisms for Wireless Body Area Networks (WBANs), with a focus on energy efficiency, adaptability, and applicability in healthcare IoT scenarios. To ensure comprehensive and reproducible coverage of the field, a structured literature search strategy was employed.

The literature search was primarily conducted using major academic databases, including IEEE Xplore, Scopus, Web of Science, PubMed, and Google Scholar. These databases were selected due to their extensive coverage of peer-reviewed publications in electrical engineering, computer science, biomedical informatics, and related interdisciplinary fields relevant to WBANs.

The publication time span was primarily restricted to works from 2014 to 2025, prioritizing recent developments while including earlier foundational contributions that remain highly influential and frequently cited as benchmarks in contemporary studies.

Studies were included if they were peer-reviewed journal articles, conference proceedings, or reputable preprints that directly proposed, analyzed, or evaluated power control mechanisms specifically for WBANs, provided quantitative results on energy efficiency, network lifetime, reliability, QoS, or adaptability in on-body, implantable, or wearable contexts, and addressed healthcare IoT applications or body-centric channel characteristics. Publications were excluded if they focused solely on general wireless sensor networks or other non-body-area systems without considering WBAN-specific challenges, if they were purely hardware-oriented without algorithmic power control contributions, or if they were non-English, non-peer-reviewed, or low-quality sources.

Additional references were incorporated through backward and forward citation tracking of highly cited included papers to capture seminal or emerging works not identified in the initial search. A total of approximately 103 papers were identified and screened, with 28 meeting the inclusion criteria and forming the core of this review.

This methodology provides a transparent and reproducible foundation for the review while allowing the flexibility needed for a narrative synthesis of a rapidly evolving interdisciplinary topic.

## 3. Power Control Mechanisms

Power control is essential for optimizing energy efficiency and ensuring QoS in wireless body area networks for healthcare Internet of Things applications. This section outlines the critical objectives of power control strategies to address energy constraints and dynamic conditions, enhancing network reliability and longevity. Power control in wireless optimizes network performance. These include minimizing energy waste to extend battery life, ensuring data reliability (e.g., maintaining target bit error rates), adapting to dynamic channel conditions (e.g., body motion, interference), balancing energy consumption with QoS requirements (e.g., low latency, high throughput), and extending network lifetime through load balancing across nodes. Collectively, these goals ensure that wireless body area networks deliver reliable, efficient, and sustainable performance for critical applications like medical monitoring.

**Figure 2:**
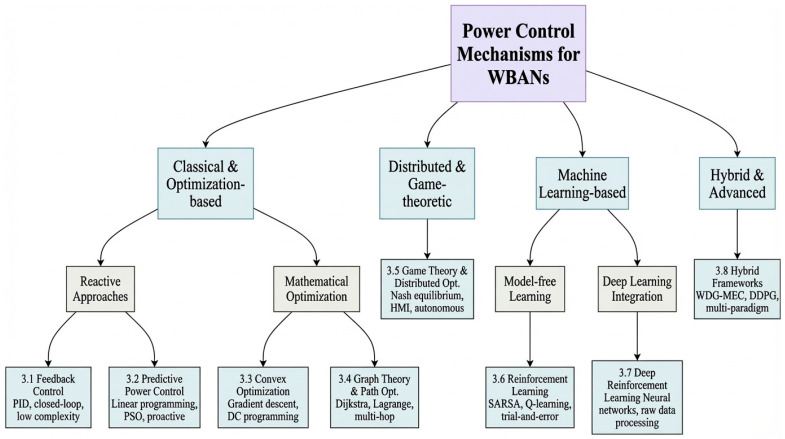
Classification Diagram of Power Control Mechanisms.

To systematically outline the various power control strategies explored in this paper, Figure 2 categorizes them into three primary groups based on core principles and design philosophies.

Classical and optimization-based methods encompass foundational and model-driven strategies, including feedback control, predictive power control, convex optimization, and graph-theoretic path optimization. These approaches typically rely on explicit mathematical models, offer provable performance guarantees, and exhibit relatively low computational complexity, making them suitable for stable or resource-constrained scenarios.

Distributed and game-theoretic approaches encompass autonomous coordination mechanisms designed for multi-node environments, such as game theory, reinforcement learning (RL), and deep reinforcement learning. These strategies enable nodes to learn, compete, or collaborate with minimal central oversight, effectively addressing interference management and scalability challenges in dynamic environments.

Hybrid and advanced methods represent the fusion of multiple paradigms (e.g., hybrid frameworks) and leverage emerging architectures (e.g., federated learning, base-stationless MIMO). They combine strengths from different categories to collectively address complex, large-scale, or privacy-sensitive deployment scenarios—situations that exceed the capabilities of any single approach.

This classification system provides a navigational guide for subsequent detailed examination, clarifying the relevance, evolutionary paths, and complementary roles of various mechanisms in addressing WBAN energy efficiency challenges.

### 3.1. Feedback Control

Feedback control is one of the most practical and widely studied power control paradigms in WBANs due to its closed-loop nature, low computational requirements, and ability to react promptly to the highly dynamic on-body channel.

The core idea of feedback-based power control is to continuously monitor real-time link quality and adjust transmission power dynamically to keep the link marginally above the required reliability threshold, thereby eliminating excessive power waste. Common feedback metrics include Received Signal Strength Indicator (RSSI), Link Quality Indicator (LQI), Packet Reception Ratio (PRR), and Signal-to-Noise Ratio (SNR).

**Figure 3:**
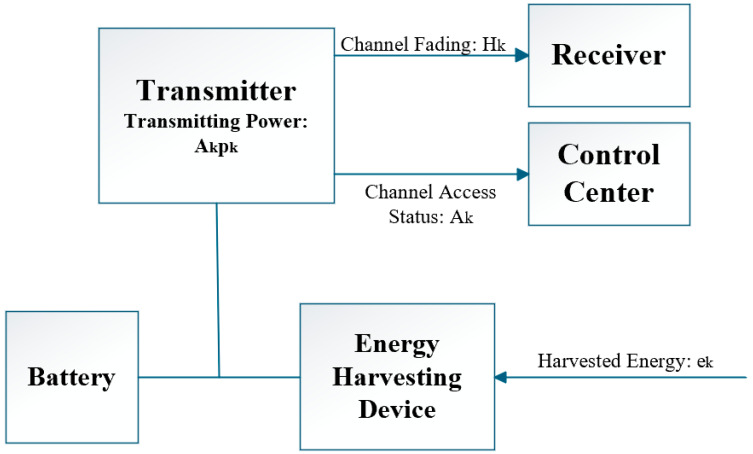
Feedback-based power control architecture for energy-harvesting WBAN systems (adapted from [7]).

Figure 3 illustrates a typical feedback control system for energy-harvesting WBANs. The transmitter dynamically adjusts its transmission power (A_k_P_k_) based on channel state information (H_k_) received from the receiver through feedback and its own energy harvesting status (e_k_). The Channel Access Control Center computes the optimal power coefficient (A_k_) using convex optimization or PID control algorithms, forming a closed-loop control system. This architecture aligns with the MDP framework proposed by Wang et al. [7], where real-time feedback of harvested energy levels and channel states enables the system to operate near the energy-neutral boundary, achieving up to 30% effective energy savings in specific scenarios (e.g., operating near the energy-neutral boundary with stable data queues) [7].

Wang et al. [7] formulated power allocation in energy-harvesting WBANs as a Markov Decision Process (MDP) and solved it using convex optimization within a feedback framework. By feeding back the current harvested energy level and channel state, the transmitter dynamically scales its transmission power. This closed-loop mechanism enables the system to operate close to the energy-neutral boundary, achieving a peak effective energy saving of 30% in scenarios with stable data queues and energy-neutral operation. This represents the peak performance reported in specific evaluated scenarios, particularly for implantable or wearable nodes powered by unpredictable sources (e.g., body heat, motion, or indoor light), rather than an average across all conditions [7].

Another important class of feedback mechanisms extends traditional industrial Proportional-Integral-Derivative (PID) controllers to wireless body area networks. These approaches treat the difference between the measured link quality (typically a weighted moving average of LQI or PRR) and the target quality as the error signal. The PID controller then computes the necessary power increment or decrement in discrete steps (usually 1–3 dBm). The proportional term provides immediate response to sudden RSSI drops caused by limb movement, the integral term eliminates steady-state error due to long-term body shadowing (e.g., arm crossing the torso), and the derivative term dampens oscillations during periodic motions such as walking or running. Real-body experiments reported in the literature show that well-tuned PID-based power control can achieve packet reception rates as high as 97.6–98.5% while reducing average transmission energy by 17–20% and extending overall network lifetime by a peak of 28% (average 22%) in posture-varying scenarios (sitting, standing, walking) compared to static or open-loop schemes. These gains are particularly pronounced in posture-varying scenarios (sitting, standing, walking), where fixed-power transmission would either waste energy when the channel is good or suffer high packet loss when the channel temporarily degrades.

The low computational overhead of PID-style feedback makes it especially suitable for resource-constrained sensor nodes that cannot afford complex optimization or learning algorithms. Moreover, the control loop can be fully distributed: each node independently adjusts its own power based on acknowledgments (or lack thereof) from the sink, requiring no global coordination or channel state broadcasting.

In summary, feedback control mechanisms, ranging from convex-optimization-assisted stochastic control [7] to lightweight PID and its variants, strike an excellent balance between implementation simplicity, responsiveness, and energy efficiency. They remain the method of choice for many practical WBAN deployments, particularly when computational resources are severely limited or when ultra-low latency adjustment is required in critical medical monitoring applications.

### 3.2. Predictive Power Control

Predictive power control represents an evolution beyond pure feedback schemes by explicitly incorporating predictive elements, real-time channel quality assessment, and traffic-aware adaptation to achieve finer-grained energy savings while maintaining strict reliability in highly variable on-body environments.

The fundamental principle is to continuously evaluate instantaneous channel conditions and traffic demands, then proactively adjust transmission power and duty cycle rather than merely reacting to past packet errors. Amjad et al. [8] demonstrated this concept in self-sustained WBANs by formulating source power optimization as a linear programming problem that minimizes transmit power subject to minimum rate constraints. By dynamically scaling power according to the latest channel quality estimates (derived from RSSI or SNR feedback) and residual battery level, the approach reduces average energy consumption by approximately 20% during dynamic physical activities such as walking, running, or sports training, with negligible degradation in packet delivery ratio. This is particularly beneficial for energy-critical nodes (e.g., implantable glucose sensors or chest-worn ECG monitors) where even small excess transmission power dramatically shortens operational lifetime.

A closely related family of dynamic techniques draws inspiration from particle swarm optimization (PSO) principles. Instead of solving a static optimization problem, these methods iteratively refine transmission power across the node population by treating each candidate power level as a particle that moves toward lower total network transmit power while satisfying individual rate requirements (typically ≥50 kbit/s for vital-sign streaming). The iterative nature allows the system to escape local minima common in rapidly changing postural channels (e.g., arm swinging across the torso), achieving stable low-energy convergence even when traditional linear programming would require frequent re-solving. The same population-based search philosophy naturally balances energy load across nodes, preventing early depletion of strategically placed relays.

In dense environments where multiple WBANs coexist (e.g., hospital wards, rehabilitation centers, or sports arenas), dynamic power control must additionally mitigate inter-WBAN interference. Game-theoretic formulations model each node or each independent WBAN as a selfish player that autonomously selects its transmission power to maximize its own utility (defined as achievable rate minus power cost). Through repeated local interactions and minimal information exchange, the system converges to a Nash equilibrium in which total transmit power is minimized while global throughput remains acceptable. This distributed convergence process effectively suppresses co-channel interference without requiring a central coordinator, making it scalable to crowded scenarios.

At the MAC layer, dynamic duty-cycle adjustment complements transmit power control. By continuously monitoring historical throughput and predicting near-future traffic patterns, the protocol increases the active duty cycle during bursts of high-priority medical data (e.g., arrhythmia alerts) to guarantee low latency, while aggressively lowering the duty cycle during periods of routine low-rate monitoring. Simulations consistently show 15–20% average reduction in overall energy consumption and a peak of 30% lower end-to-end latency under bursty traffic, demonstrating that joint dynamic management of radio-on time and transmission power yields multiplicative rather than merely additive gains.

The effectiveness of power control is inherently coupled with the underlying medium access control (MAC) strategy, which governs channel access and collision avoidance. In TDMA-based schemes, deterministic time slots eliminate contention and packet collisions, allowing power control to focus on compensating channel fading with minimal interference-induced uncertainty. This enables precise, low-margin power adjustment and stable energy prediction. In contrast, CSMA/CA-based protocols introduce probabilistic channel access, where collisions and subsequent retransmissions become significant energy drains. Here, power control must not only adapt to channel conditions but also account for varying contention levels and the extra energy cost of backoff and retransmission.

In summary, dynamic power control, as exemplified by linear programming-based adaptation [8], PSO-inspired iterative refinement, game-theoretic interference coordination [9], and traffic-aware duty-cycle scheduling, moves beyond reactive feedback toward proactive, context-aware energy management. These mechanisms are especially powerful in scenarios dominated by unpredictable body motion, variable data rates, and coexisting networks, consistently delivering 15–20% energy savings while preserving or even improving end-to-end performance compared to static or purely feedback-driven approaches.

### 3.3. Convex Optimization

Convex optimization has emerged as a cornerstone for power control in WBANs because many practical sub-problems (or their tight approximations) can be cast into convex forms that admit globally optimal, low-complexity solutions even on resource-limited nodes.

Hakami and Dehghan [10] formulated distributed power control for energy-harvesting cooperative relay networks as a delay-minimization problem and solved it through asynchronous gradient descent on a convex surrogate. By exploiting the convexity of the objective, nodes update their power policies using only local channel and battery information plus minimal signaling, achieving near-optimal end-to-end delay (within 15% of the theoretical lower bound) while reducing signaling overhead by more than 90% compared with centralized schemes. This makes the approach highly suitable for real-time telemetry in mobile patients where both energy and latency are critical.

Subsequent works further extend convex formulations to joint power control and computation offloading in dense WBANs. Non-convex constraints arising from body-induced fading and task deadlines are successively approximated into convex sub-problems via difference-of-convex (DC) programming and successive convex approximation (SCA). The resulting iterative algorithm yields up to 13.8% reduction in total energy consumption and 14.3% extension of network lifetime in cross-layer designs that simultaneously account for channel variability, energy harvesting uncertainty, and QoS requirements of non-emergency physiological monitoring.

Another important convex approach employs sigmoidal utility functions to reflect the priority differences among heterogeneous medical traffic (e.g., critical ECG vs. routine temperature). By incorporating instantaneous channel states and queue waiting times into the utility, the original non-convex resource allocation problem is transformed into a tractable difference-of-convex (DC) program. Solving it via the convex-concave procedure (CCCP) or dedicated DC algorithms (DCA) achieves significant throughput gains of up to 35% (equivalent to 23 Mbps in typical medical monitoring scenarios) while simultaneously extending network lifetime by an average of 14% in typical medical monitoring scenarios.

In summary, convex optimization techniques—including distributed gradient-based delay minimization [10], successive convex approximation for joint power-task offloading [11], and DC programming with sigmoidal utilities—transform otherwise intractable non-convex problems into efficiently solvable forms. They consistently deliver 13–14% energy savings and lifetime improvements with provable convergence and low overhead, making them particularly attractive for stable or slowly varying WBAN deployments where global optimality and theoretical guarantees are more important than ultra-fast adaptation to extreme dynamics.

### 3.4. Graph Theory and Path Optimization

Graph theory provides a natural and powerful modeling framework for multi-hop and relay-assisted WBANs, where direct links to the sink are often blocked or highly attenuated due to body shadowing. By explicitly representing nodes, residual energy, and dynamic link quality as vertices and weighted edges, graph-based power control transforms routing and power allocation into joint optimization problems that simultaneously maximize reliability and minimize total transmission energy.

In energy-harvesting WBANs, battery states are first discretized into finite levels, and possible transitions are modeled as a Markov chain. The resulting weighted directed graph assigns edge costs that reflect both current link quality (derived from RSSI in hello packets) and predicted outage probability. Traditional shortest-path algorithms, such as Dijkstra, are then significantly enhanced to search not for minimum hop count, but for the path that maximizes end-to-end throughput under strict power and latency constraints. Multi-constrained optimization is efficiently handled through Lagrange duality, yielding the optimal transmission power and scheduling instant for each hop and achieving 35–47% higher network-wide throughput compared with conventional minimum-hop routing protocols that use fixed transmission power.

A complementary graph-theoretic technique exploits the classic Friis transmission equation to compute the minimum required transmission power for each neighbor directly from measured RSSI in periodically exchanged hello packets. The calculated power values are stored in a local neighbor table. When selecting the next hop, a probabilistic forwarding strategy jointly considers the neighbor’s residual energy and the required transmission power: nodes with higher remaining energy and lower required power are preferred. This simple yet effective policy significantly improves energy balance across the network, reduces the probability of early node failure, and extends overall lifetime, especially in large-scale or multi-hop implantable-to-surface scenarios.

In summary, graph-based approaches—including Markov-modeled weighted digraphs with enhanced Dijkstra and Lagrange-based multi-constrained optimization, combined with Friis-derived minimum-power neighbor tables and energy-aware probabilistic forwarding—offer a systematic way to achieve global energy efficiency in multi-hop WBANs. They consistently deliver 35–47% throughput improvement (versus conventional minimum-hop routing with fixed power) and markedly better energy balance without introducing excessive computational overhead on sensor nodes.

### 3.5. Game Theory and Distributed Optimization

Game theory and distributed optimization provide elegant solutions for WBANs where nodes are selfish, lack a central controller, or need to coordinate under incomplete information. By modeling each sensor or entire WBAN as an independent player that maximizes its own utility, these approaches naturally achieve globally desirable outcomes such as minimal total transmission power and fair energy consumption.

Non-cooperative game frameworks combined with Hidden Markov Models (HMM) are used to predict time-varying on-body channel states without requiring perfect instantaneous knowledge. Each node treats the channel as a hidden state and updates its belief through local observations. The resulting power selection game converges significantly faster (28% fewer iterations) than pure non-cooperative games without prediction, while reducing excess margin in signal-to-noise ratio, leading to 15% higher energy utilization efficiency and 27% longer network lifetime.

In software-defined WBANs, the network is represented as an undirected graph where edge weights reflect distance, residual energy, and hop count. A centralized controller (logically centralized but physically running on the sink or hub) iteratively adjusts the utility function of a non-cooperative game until all players reach a Nash equilibrium. This global view enables better load balancing, eliminating unnecessary movement redundancy in coverage and extending network lifetime by approximately 30% while reducing redundant transmissions by 25%.

Another powerful distributed paradigm is hybrid scheduling plus local power control. Time slots are first allocated centrally according to traffic priority and queue length, whereas transmission power within each allocated slot is adjusted locally via cross-layer information (channel state, residual energy, interference level). The joint objective maximizes weighted sum-rate minus power cost. Thanks to the separation of scheduling and power dimensions, the distributed algorithm reduces total energy consumption by up to 30% while maintaining excellent delay-energy balance even in large-scale, dense deployments.

In summary, game-theoretic and distributed optimization approaches—ranging from HMM-enhanced non-cooperative games, software-defined Nash equilibrium iteration, to hybrid centralized scheduling with distributed power control—enable fully autonomous or semi-autonomous operation. They consistently deliver 27–30% lifetime extension, faster convergence, and robust performance in multi-node, interference-limited, or controller-less WBAN scenarios.

### 3.6. Reinforcement Learning

Reinforcement learning (RL) enables WBAN nodes to gradually discover optimal power control policies through trial-and-error interaction with the highly uncertain on-body environment, without requiring explicit channel models or future knowledge.

Masadeh et al. [12] applied the classic SARSA algorithm to energy-harvesting communication systems. Using ε-greedy exploration, the agent learns to map current battery level, harvested energy, and observed channel quality to the best transmission power. The learned policy achieves 15% higher average data rate compared with model-based baselines and reaches 95% of the theoretical optimum even under highly fluctuating energy income and body-induced fading, making it ideal for long-term environmental sensing nodes that cannot rely on accurate prediction models.

Nasreen et al. [13] proposed a Q-learning-based energy-efficient power allocation framework (Q-EEPA) for Wireless Body Area Networks (WBANs), focusing on tier 1 and tier 2 communications. The Q-learning algorithm is employed to jointly allocate transmission power levels and select routing paths/next hops, with inputs incorporating heterogeneous wearer activities and outputs from extensive game theory to handle both interference and non-interference scenarios. The approach aims to achieve a high packet delivery ratio with minimum power levels, minimum hop counts, and effective interference mitigation, thereby reducing energy consumption while improving network performance. This framework is suitable for energy-constrained WBANs in medical monitoring applications, complementing classical feedback control and optimization-based methods, particularly under dynamic channel conditions.

To handle large-scale coexisting WBANs, mean-field multi-agent RL (MF-MARL) [14] treats the influence of numerous neighboring networks as an averaged “mean field” rather than tracking every individual state. Each agent approximates its own Q-function using deep neural networks trained in a fully distributed manner, eliminating the need for explicit state sharing among agents. This mean-field approximation significantly reduces communication overhead compared to centralized deep RL approaches (e.g., by obviating the need for global state exchange), making the method scalable to crowded environments (e.g., smart gyms or hospital wards). Although this introduces a minor trade-off in optimality compared to full multi-agent coordination, this scalability gain comes at the cost of approximating full multi-agent state information, resulting in near-optimal rather than globally optimal solutions (e.g., potentially achieving near-maximum rather than absolute maximum aggregate network throughput), it still maintains robust performance in high-density scenarios.

Multi-agent actor-critic extensions further jointly optimize duty cycle and transmission power by observing only local buffer occupancy and battery charge. Thanks to continuous action spaces and shared critic networks, the learned policies exhibit near-zero system collapse probability over multi-year horizons, significantly outperforming traditional estimation-based adaptation schemes in both harvested energy stability and end-to-end throughput.

In summary, reinforcement learning—from single-agent SARSA [12] and Q-learning-based energy-efficient power allocation [13] to scalable mean-field MARL [14] and actor-critic duty-power co-optimization—offers model-free, long-term optimal adaptation. It consistently delivers 15–18% energy reduction and superior robustness, with MF-MARL particularly addressing the scalability challenge by reducing communication overhead at the cost of only negligible throughput degradation in large-scale settings.

### 3.7. Deep Reinforcement Learning

Deep reinforcement learning (DRL) extends classical RL by replacing tabular value functions with deep neural networks, enabling WBAN nodes or hubs to handle high-dimensional raw observations (channel traces, traffic patterns, accelerometry, battery curves) and continuous power spaces that are far beyond the reach of traditional RL.

**Figure 4:**
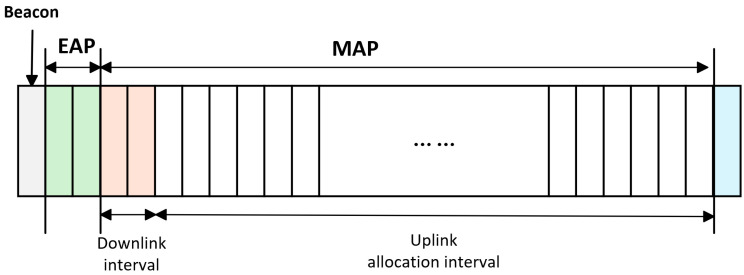
Superframe structure of the IEEE 802.15.6 standard.

Kim et al. [15] pioneered DRL integration with IEEE 802.15.6-standard scheduling, leveraging its superframe structure—comprising beacon, random access (RAP), exclusive access (EAP), and managed access phases (MAP), as illustrated in Figure 4. Their DRL agent processes flow-level features (packet rate, priority) and real-time channel statistics as state inputs, then jointly optimizes transmission power and user scheduling. Through a carefully designed reward function balancing reliability, QoS, and energy, they achieved 15% lower end-to-end latency while maintaining high packet delivery ratios. This DRL approach proves particularly valuable for personalized chronic disease management, where critical alarms must be dynamically prioritized over routine data.

The integration of DRL with this superframe structure enables intelligent, context-aware resource allocation. The DRL agent observes dynamic network conditions (e.g., channel quality, traffic priority, and buffer states) at the start of each superframe and then jointly determines transmission power and user scheduling per phase. For instance, during the EAP phase—designated for high-priority or emergency traffic—the agent can allocate higher transmit power to guarantee reliability and low latency. In the contention-based RAP or scheduled MAPs, power levels can be adaptively reduced for routine data or optimized to mitigate collisions and interference. By learning to map specific superframe intervals to appropriate power-scheduling actions, DRL effectively unifies PHY-layer power control with MAC-layer time-resource management, leading to enhanced energy efficiency and QoS in dynamic WBAN environments.

In high-density or interference-heavy environments, hybrid DRL-MEC frameworks offload the neural network inference and occasional retraining to a mobile edge server, while keeping lightweight experience collection on the sensor side. By combining offline pre-training with online fine-tuning, the policy dynamically adjusts transmission power to combat inter-WBAN interference, achieving up to 15% higher aggregate data rates compared with pure online DRL or conventional schemes [16].

Age-of-Information (AoI)-aware power control further demonstrates DRL’s strength in timeliness-critical medical applications. Using neural basis expansion analysis (NBEATS) inside the DRL loop for short-term channel prediction, the agent proactively lowers power when the channel is predicted to be good and increases it only when necessary, reducing average AoI by 7% and energy by 6% relative to reactive baselines.

Hybrid supervised + DRL schemes first train a supervised predictor on historical channel traces to estimate future fading, then feed the prediction into a DRL agent that selects both transmission power and block length for relay-assisted links. The resulting policy approaches the performance of exhaustive search while maintaining stable operation across highly dynamic walking and running postures.

In summary, deep reinforcement learning—whether standalone DRL scheduling under 802.15.6 [15], hybrid DRL-MEC for dense networks [16], AoI-aware predictive control, or supervised-DRL channel forecasting—excels at processing raw, high-dimensional sensory data and optimizing continuous actions. It consistently delivers 6–15% gains in latency, energy, or freshness, making it the method of choice for next-generation WBANs that demand both extreme adaptability and handling of complex, multi-modal inputs.

### 3.8. Hybrid Frameworks

Hybrid frameworks deliberately combine the strengths of multiple paradigms (optimization, machine learning, edge computing, and traditional heuristics) to overcome the individual limitations of any single method, thereby achieving higher performance in dense, dynamic, and heterogeneous WBANs.

Su et al. [17] proposed a wind-driven optimization (WDO) algorithm tightly coupled with mobile edge computing (MEC). The edge server periodically collects channel and battery states from all nodes, runs the population-based WDO to solve the global power allocation problem, and then pushes the near-optimal power vector back to sensors. This hybrid approach exploits the strong global search ability of WDO and the abundant computation power of the edge, delivering approximately 20% higher network throughput and 30% lower total energy consumption in dense deployments compared with purely local or purely centralized baselines.

Li et al. [18] developed a hybrid supervised/reinforcement learning framework that first employs a classification network to predict whether transmission should occur based on minimal packet error probability (avoiding energy waste on poor channels), then uses a deep reinforcement learning (DRL) actor network to jointly determine optimal transmit power levels and blocklength values for the current session. The combination yields near-optimal energy consumption (almost identical to exhaustive search) in highly dynamic WBAN environments while maintaining low computational burden suitable for wearable/relay nodes.

Rajawat et al. [9] designed an AI-based power-saving protocol that integrates hidden Markov models for channel prediction with reinforcement learning for long-term policy refinement, further augmented by an intelligent sleep mechanism that drastically reduces idle listening. The resulting multi-layer hybrid significantly improves end-to-end delay, energy efficiency, and overall network lifetime in dense scenarios.

Another representative hybrid is the Power Control and Task Offloading (PCTO) strategy based on Deep Deterministic Policy Gradient (DDPG) [19]. The DDPG agent treats joint power selection and computation offloading as a continuous-action Markov Decision Process, accelerated by prioritized experience replay. The multi-objective reward carefully balances transmission delay, energy consumption, and SINR under strict QoS constraints. In 50-node simulated dense WBANs, it achieves 25% lower task latency and 20–30% higher overall satisfaction compared with single-technique benchmarks.

Additional multi-technology fusions include distributed scheduling with fairness indices combined with neural network-based adaptive power control, as well as SPIDNN structures that react to link changes in real time. These hybrids maintain packet delivery ratios above 96.5% while reducing total energy by 17% and extending lifetime by 20%.

In summary, hybrid frameworks—whether WDO + MEC [17], hybrid supervised + deep reinforcement learning [18], HMM + RL + sleep [9], DDPG-based joint power-offloading [19], or fairness-aware neural scheduling—consistently push the performance envelope, routinely achieving 17–30% energy reduction, higher throughput, lower latency, and better fairness in the most challenging dense and dynamic WBAN environments.

### 3.9. Emerging Architectures

Emerging architectures go beyond traditional single-controller WBANs by introducing fundamentally new network topologies, multi-modal sensing, privacy-first learning, and forward-looking communication paradigms to meet the extreme requirements of future medical and consumer applications.

Cell-free massive MIMO architectures eliminate conventional small-cell boundaries: multiple distributed access points cooperate via backhaul to serve all body sensors simultaneously. Throughput-equalization power control (TEPC) [20] dynamically adjusts the power coefficients of all access points so that every sensor experiences similar uplink/downlink quality regardless of its position on the body. This cooperative approach dramatically reduces deep fading caused by body blockage and improves both average spectral efficiency and fairness by 25% compared with traditional single-access-point designs.

Kinematics and biosignal-assisted architectures [21] continuously collect accelerometer, gyroscope, and even heart-rate data to predict the near-future on-body channel state. The predicted channel is then used for proactive packet scheduling and transmission power reduction. In daily activities (walking, sitting, running), this multi-modal prediction reduces average required transmission power by 27% and increases average packet delivery ratio by 41%, making it particularly effective for ambulatory elderly monitoring where body motion is highly unpredictable.

Privacy-preserving federated learning [16,22] enables multiple WBANs or devices to collaboratively train a shared power-control model without ever exchanging raw physiological data. Only model gradients or updates are transmitted, achieving up to 25% energy savings in dense telemedicine deployments while fully protecting patient privacy. This architecture is especially critical for sensitive medical scenarios where data leakage is unacceptable.

Large-language-model-driven cognitive control planes [23] treat the entire WBAN as a reasoning system: an LLM continuously processes multi-modal telemetry (battery level, harvesting rate, channel state, motion patterns) and outputs high-level decisions such as switching between RF and human-body communication, adjusting PHY modes, or activating intelligent reflecting surfaces (IRS). Early conceptual studies suggest potential energy savings of 30–50% for ultra-reliable low-latency chronic disease monitoring in 6G-ready networks.

Lightweight AI-enhanced intrusion detection [23] runs on edge nodes and thresholds abnormal power consumption spikes (>0.5 J) that may indicate denial-of-service or replay attacks. By combining anomaly detection with immediate power throttling, it achieves 95% detection accuracy while saving an additional 20% energy in remote healthcare deployments.

In summary, emerging architectures—cell-free cooperative MIMO with TEPC [20], kinematics + biosignal prediction [21], privacy-first federated learning [22,23], LLM cognitive control [23], and AI-based security [23]—represent the future direction of WBANs. They consistently deliver 20–50% energy reduction, dramatically higher reliability, and built-in privacy/security, paving the way for truly scalable, secure, and intelligent body area networks in healthcare IoT.

### 3.10. Taxonomy and Decision Framework for Power Control Mechanism Selection

**Figure 5:**
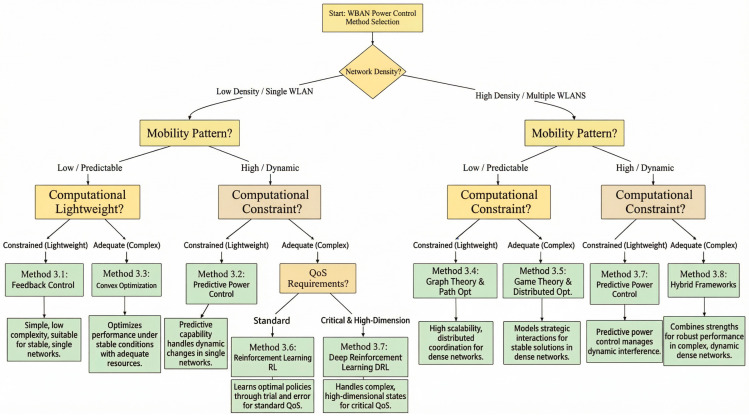
A decision-flow taxonomy for selecting WBAN power control mechanisms based on network density, mobility pattern, and computational constraints.

Having reviewed the diverse power control mechanisms in Section 3.1, Section 3.2, Section 3.3, Section 3.4, Section 3.5, Section 3.6, Section 3.7, Section 3.8 and Section 3.9, it is evident that no single approach is universally optimal. The efficacy of a method is highly contingent upon specific deployment scenarios and system constraints. To synthesize this knowledge and provide a practical guide for selecting appropriate mechanisms, we propose a multi-dimensional decision-flow taxonomy, as illustrated in Figure 5.

This framework operates based on three critical, application-dependent dimensions:

Network Density: Distinguishes between isolated/single-WBAN deployments and dense multi-WBAN scenarios. The latter introduces significant challenges in inter-network interference and coordination overhead, directly impacting scalability—a key issue to be further analyzed in Section 3.
Mobility Pattern: Categorizes scenarios based on the predictability and dynamics of node movement, ranging from low/static (e.g., post-operative monitoring) to high/dynamic (e.g., athlete training). This dimension critically affects the choice between reactive and predictive/prescriptive strategies.
Computational Constraints: Separates resource-constrained sensor nodes, which mandate lightweight algorithms, from more powerful coordinators or edge servers capable of supporting complex optimizations or AI-driven solutions.

As depicted in the flowchart, this taxonomy logically maps specific technical challenges (e.g., need for distributed coordination in dense networks, requirement for channel prediction under high mobility) to the families of algorithms reviewed earlier. For instance, Game-Theoretic approaches (Section 3.5) become relevant for distributed interference management in dense deployments, while Deep Reinforcement Learning (Section 3.7) is a candidate for complex, dynamic environments with adequate computational resources. This structured perspective not only summarizes the reviewed techniques but also clearly highlights the unresolved trade-offs and scenario-specific challenges, forming a natural bridge to the discussion of open research problems in the following section.

### 3.11. Comparative Analysis of Power Control Protocols

This subsection compares representative power control protocols for WBANs, focusing on energy savings, adaptability, and applicability, based on cited studies. Optimization-based methods offer efficient solutions for stable, low-complexity scenarios, providing reliable performance with minimal overhead. Learning-based approaches, including RL and DRL, enhance adaptability to dynamic conditions, supporting critical WBAN applications. Hybrid and emerging frameworks, such as WDO with MEC and federated learning, address dense and privacy-sensitive environments, improving throughput and data security.

Table 1 summarizes the performance metrics, strategies, and testing scenarios of selected protocols, providing a quantitative comparison to highlight their strengths and trade-offs.

**Table 1:** Comparative analysis of representative power control protocols.

Reference	Strategy Type	Energy Savings	Testing Scenario	Applicable Scenarios	Complexity
Wang et al. [7], 2014	Feedback Control (MDP + Convex Optimization)	Up to 30% effective energy savings	Energy-harvesting WBANs with causal channel and harvesting info; fading channels	Implantable/wearable nodes with unpredictable energy sources (e.g., body heat, motion)	L
Amjad et al. [8], 2019	Dynamic Power Control (Linear Programming)	Approximately 20% energy reduction	Self-sustained WBANs; dynamic physical activities (walking, running, sports training)	Energy-critical nodes (e.g., implantable glucose sensors, chest-worn ECG monitors)	L
Rajawat et al. [9], 2022	Hybrid (Hidden Markov Models + RL)	Not quantified; improved efficiency via intelligent sleep mechanism	Dense WBANs; simulations for medical monitoring	General medical monitoring in dense settings	H
Hakami and Dehghan [10], 2016	Convex Optimization	Near-optimal delay (within 15% of the lower bound); over 90% reduction in signaling overhead	Energy-harvesting cooperative relay networks; bursty data arrival	Real-time telemetry in mobile patient monitoring	M
Masadeh et al. [12], 2018	Reinforcement Learning	Improved efficiency (higher data rates)	Energy-harvesting communications systems	Dynamic harvesting environments with no prior knowledge	H
Kim et al. [15], 2022	Deep Reinforcement Learning	15% energy reduction, 15% latency reduction	Personalized WBANs; chronic disease monitoring (e.g., diabetes tracking)	Chronic care & personalized health tracking	H
Xu et al. [16], 2022	Deep Reinforcement Learning	Energy minimization; up to 15% higher data rates	Wirelessly powered IoT sensors; session-specific	Data collection from wirelessly powered sensors under latency constraints	H
Su et al. [17], 2024	Hybrid (Wind-Driven Optimization + MEC)	30% energy savings, 20% throughput increase	Dense networks; multi-user fitness tracking	Fitness tracking & multi-user environments	H
Liao et al. [19], 2025	Hybrid (DDPG-based)	20–30% energy savings; 25% lower latency	High-density WBANs; multi-user simulations	Dense WBAN environments with MEC offloading	H
Bao et al. [20], 2024	Throughput-Equalization Power Control	~25% performance improvement (indirect energy benefits)	Multi-node hospital settings; cell-free model simulations	Hospital-based multi-patient monitoring	H
Benabderrahmane et al. [24], 2026	Federated Learning	Reduced communication overhead & latency via edge processing (manageable for resource-constrained devices)	Privacy-preserving remote patient monitoring	Privacy-sensitive remote monitoring	H
Mohammadi et al. [25], 2024	Deep Reinforcement Learning	49.95% reduction in sampling rate & 89.7% in unnecessary transmissions (achieving self-sustainability)	Intelligent WBANs; energy-harvesting optimization	Ambient/environment-aware sensing & monitoring	H

Abbreviations: RL—Reinforcement Learning, MEC—Mobile Edge Computing. Testing conditions include: (1) dynamic body motion (walking, running, arm swing), (2) dense multi-WBAN coexistence (≥3 networks), (3) energy harvesting from ambient sources (RF/thermal/kinetic), and (4) medical-grade traffic (ECG, SpO_2_) with strict QoS requirements. All results are based on simulation or experimental studies cited in the corresponding references. Complexity levels: L (Low), M (Medium), H (High), corresponding to computational and implementation complexity as categorized in Table 2.

**Table 2:** Complexity Analysis of Power Control Types.

Power Control Type	Computational Complexity	Scalability (Nodes)	Memory Requirement	Convergence Time
Feedback Control	Low (simple arithmetic/PID operations)	Medium-High (distributed, but interference may limit dense scaling)	Low (stores few state variables)	Fast (immediate reaction per control cycle)
Predictive Power Control	Medium (adds prediction/optimization step)	Medium (coordination needed for interference prediction)	Medium (requires history for prediction)	Medium (depends on prediction horizon & update rate)
Convex Optimization	Medium-High (solving convex problems iteratively)	Low-Medium (centralized or requires signaling for distributed versions)	Medium (stores optimization variables & channel info)	Medium-Slow (iteration-dependent, slower for distributed cases)
Graph Theory and Path Optimization	Medium-High (pathfinding algorithm overhead)	High (inherently handles multi-hop networks)	Medium-High (stores graph topology & link states)	Slow (re-convergence needed on topology change)
Game Theory and Distributed Optimization	Medium (local utility computation & iteration)	High (designed for distributed, selfish nodes)	Low-Medium (local state & neighbor info)	Slow (iterative convergence to equilibrium)
Reinforcement Learning	Medium (value table updates or function approximation)	Low-Medium (state space grows with nodes)	Medium (stores policy/Q-table or small neural network)	Very Slow (requires extensive exploration/training)
Deep Reinforcement Learning	Very High (neural network inference/ training)	Medium (can scale with centralized/edge trainer)	High (stores deep neural network)	Very Slow (lengthy training, fine-tuning possible)
Hybrid Frameworks	High (combination of multiple techniques)	Medium-High (depends on composition, often edge-assisted)	Medium-High (components of combined methods	Variable (depends on the slowest component)
Emerging Architectures	Very High (e.g., FL, cell-free MIMO coordination)	High (designed for large-scale, dense scenarios)	High (model parameters, global views)	Slow-Variable (FL rounds, centralized optimization)

**Figure 6:**
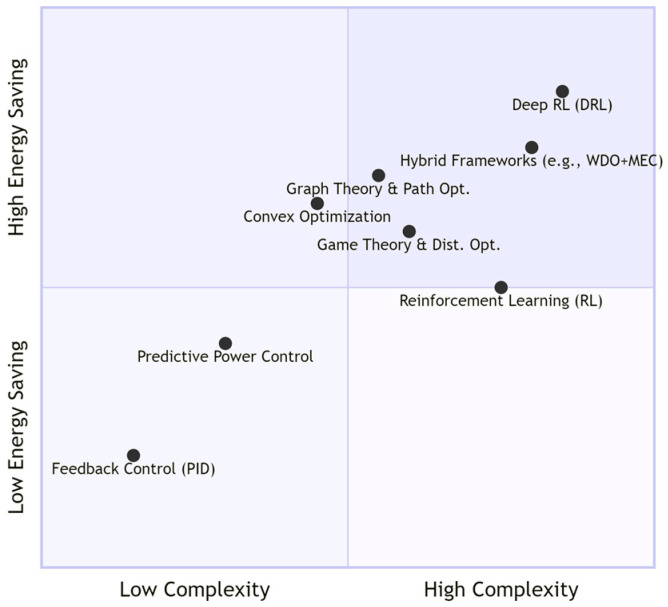
Energy Consumption vs. Complexity Tradeoff Diagram.

Figure 6 plots the evaluated power control mechanisms as a scatter diagram across energy consumption and computational complexity dimensions. In the figure, classical feedback control and predictive power control occupy the lower-left quadrant, achieving moderate energy savings with low computational overhead and fast response times. These are particularly suitable for resource-constrained sensor nodes. Methods such as convex optimization and graph-theoretic optimization occupy the middle region, achieving higher energy efficiency at the cost of moderate complexity, typically requiring more centralized coordination or signal transmission. Advanced learning-based and hybrid strategies, such as deep reinforcement learning (DRL) and hybrid frameworks, cluster in the upper-right quadrant. These achieve the greatest energy savings and adapt to complex, dense scenarios but consume substantial computational resources, memory, and convergence/training time, often requiring edge server support. This visualization reveals a key axiom in WBAN power control design: improvements in energy efficiency and adaptive intelligence typically come with increased algorithmic complexity and resource demands.

### 3.12. Failure Modes and Practical Limitations of Power Control Mechanisms

While each class of power control mechanisms offers distinct advantages, their practical deployment is bounded by specific failure modes and inherent limitations. A critical understanding of these constraints is essential for robust system design and informs the open challenges discussed in Section 4. The key limitations are synthesized below.

- Feedback Control: In rapidly changing channels (e.g., during running), high-gain PID controllers can overreact, causing power to oscillate and increasing energy waste. Feedback relies on outdated channel measurements. If body motion changes faster than the control loop update interval, transmission may operate at inappropriate power levels, leading to packet loss. Furthermore, purely reactive feedback cannot anticipate impending deep fading, leading to unavoidable communication interruptions during abrupt posture changes (e.g., when an arm crosses the torso).
- Predictive Power Control: Predictions based on linear or particle swarm optimization (PSO) assume smooth channel variations. However, sudden occlusions caused by clothing or environmental obstacles frequently violate this assumption, leading to miscalculated power requirements. Meanwhile, continuous channel estimation and traffic prediction consume additional energy and computational resources, potentially offsetting the energy savings achieved through active adjustments. Furthermore, in dense multi-wireless body area network environments, independent predictions by each network may lead to conflicting power adjustments, exacerbating rather than mitigating interference.
- Convex Optimization: Convex optimization requires problems to be convex or well-approximated, yet real human body channels often exhibit non-convexity and discontinuity. Convex approximations may converge to suboptimal or infeasible power allocation schemes. Simultaneously, iterative algorithms like gradient descent or sequential convex approximation suffer from insufficient convergence speed during rapid human movement, leading to outdated power decisions during critical monitoring periods. Furthermore, most convex optimization models require global information support, while distributed versions face the dilemma of increased signaling overhead and reduced optimality.
- Graph Theory and Path Optimization: Graph-based multi-hop paths optimized for energy efficiency are vulnerable to single-node failures (e.g., relay nodes running out of power), leading to path reconstruction overhead and service interruptions. Simultaneously, edge weights based on RSSI or remaining energy neglect time-varying interference, causing severe packet loss shortly after path selection. Furthermore, running Dijkstra or Lagrange solvers on sensor nodes may exceed their processing capacity, particularly in large or mobile networks.
- Game Theory and Distributed Optimization: Selfish nodes may form a globally suboptimal Nash equilibrium, leading to overall energy waste in the network. Simultaneously, in games based on hidden Markov models, erroneous belief updates triggered by noisy observations may force nodes to adopt catastrophically low-power modes, disrupting network connectivity. Furthermore, distributed iteration requires extensive message exchange, and network states in mobile scenarios may change before convergence is achieved.
- Reinforcement Learning: The state-action space in reinforcement learning grows exponentially with the number of nodes or WBANs, making basic reinforcement learning difficult to handle in dense deployments. Simultaneously, learning rates are slow in non-stationary environments, where airborne channels may have already changed, rendering learned policies ineffective. Furthermore, agents may select excessively high or low power levels during necessary exploration phases, leading to packet bursts or losses—particularly dangerous in critical monitoring scenarios.
- Deep Reinforcement Learning: DRL policies trained on specific motion patterns (e.g., walking) may fail when encountering unknown activities (e.g., cycling), resulting in insufficient generalization capabilities. Simultaneously, constrained by memory and energy consumption, running deep neural networks on wearable or implantable nodes is often impractical, while edge offloading introduces latency. Furthermore, online fine-tuning in dynamic environments may cause neural networks to forget previously learned policies, undermining long-term performance stability.
- Hybrid Frameworks: Integrating multiple algorithms (e.g., WDO + MEC [17]) introduces additional hyperparameters and failure points, increasing tuning complexity and reducing deployment robustness. Simultaneously, hybrid architectures combining centralized and distributed components require precise time synchronization, while edge-server update delays can cause misalignment in local decision-making.
- Emerging Architectures: Unitless MIMO [20] requires high-precision synchronization and stable backhaul links; federated learning [22] suffers from model divergence caused by heterogeneous user data; while LLM-driven control [23] is prone to generating physically infeasible decisions (“hallucinations”) and lacks real-time operation capabilities on wearable devices.

### 3.13. Privacy Considerations in Power Control Strategies

Privacy represents a critical and multifaceted challenge in WBANs, as sensitive physiological data and network operating patterns—such as transmission power levels and channel state information—can be exploited to infer users’ health status, activities, or location. Power control strategies impact privacy through multiple avenues: they generate observable patterns in channel access and power adjustments; optimization processes may require sharing channel or node state information; and they often rely on centralized or federated learning architectures that carry data leakage risks. This section focuses on analyzing major privacy risks and technical countermeasures in power control, with particular emphasis on their impact on energy efficiency.

- Privacy Risks in Traditional Power Control: Most classical power control mechanisms (e.g., feedback control, convex optimization, and basic reinforcement learning, RL) inherently expose privacy-sensitive information. These approaches typically require frequent exchange of channel state information (CSI), signal-to-noise ratio (SNR) reports, or packet reception acknowledgments. An eavesdropper can infer human movement, posture changes, or even specific activities (e.g., walking versus stationary) by analyzing this data. Similarly, centralized optimization frameworks create single-point data leakage risks when coordinators or cloud servers collect global network states or raw training data. Even distributed approaches relying on iterative local information exchange may expose long-term node behavior and energy consumption patterns—features correlated with health events (e.g., increased transmission rates due to elevated heart rate).
- Emerging Privacy-Preserving Technologies and Their Integration: Recent research proposes several privacy-aware mechanisms integrable with WBAN power control. Federated learning (FL), as a key framework, enables collaborative training of shared power control models by having nodes exchange only model updates (gradients), while raw physiological and channel data remain permanently local. While this prevents direct data leakage, secure aggregation of updates remains necessary, along with safeguards against inference attacks targeting gradients themselves. Differential privacy (DP) formally constrains attackers’ ability to infer individual contributions by adding calibrated noise to locally shared parameters (e.g., gradient updates or power adjustment decisions) before transmission. DP is applicable to FL and distributed optimization but introduces trade-offs between privacy strength and model accuracy/convergence speed. Homomorphic encryption (HE) enables computation directly on encrypted data (e.g., power allocation optimization), achieving privacy-preserving centralized coordination without decrypting sensitive inputs. However, current HE technologies impose prohibitively high computational and communication overhead for resource-constrained sensors. For instance, homomorphic encryption typically introduces computational overhead on the order of several magnitudes higher than plaintext operations on resource-constrained devices, resulting in significantly increased single-node energy consumption that may potentially offset efficiency gains from power control mechanisms. Secure Multi-Party Computation (SMPC) and lightweight secure aggregation protocols offer compromises, enabling groups of nodes to collaboratively compute optimal power settings without revealing individual inputs. Nevertheless, these approaches still require a non-negligible number of communication rounds.
- Energy Overhead and Practical Tradeoffs: Integrating privacy protection inevitably increases energy consumption, necessitating careful balancing against security gains. Technologies like federated learning and differential privacy primarily increase communication energy consumption due to repeated transmission of model updates or noise perturbation parameters. Homomorphic encryption and secure multi-party computation significantly elevate computational energy consumption on sensor nodes due to intensive cryptographic operations. For instance, power control based on homomorphic encryption can substantially increase single-node energy consumption compared to plaintext optimization, often negating the energy savings achieved through power control itself. Therefore, practical deployments require lightweight approaches (e.g., partial homomorphic encryption, selective differential privacy) or offloading high-energy privacy computations to more powerful edge hubs. Hybrid approaches—such as combining federated learning with distributed learning and lightweight differential privacy that protects gradients while concentrating homomorphic encryption-based coordination operations on edge servers—offer a viable path balancing strong privacy protection with acceptable energy consumption limits. This provides a feasible solution for ensuring the long-term stable operation of wireless body area networks.

### 3.14. Cross-Layer Optimization

Power control does not operate in isolation; its effectiveness is inherently tied to decisions made across the protocol stack. Cross-layer optimization systematically exchanges state information—such as channel conditions (PHY), queue lengths and access schedules (MAC), and routing paths (network)—to jointly determine transmission parameters, thereby overcoming the limitations of isolated, layer-specific designs. For instance, coupling power control with MAC scheduling can reduce idle listening and collision-induced retransmissions, while coordinating with network-layer routing can balance energy consumption across multi-hop paths. Representative approaches include joint power-and-duty-cycle optimization, power-aware routing protocols, and PHY-MAC utility maximization frameworks. Although cross-layer designs promise significant gains in energy efficiency, reliability, and latency, they introduce increased design complexity, reduced modularity, and challenges in standardization.

## 4. Challenges and Future Directions

Although significant progress has been made in power control mechanisms for Wireless Body Area Networks (WBANs), several critical unresolved research problems persist that limit their widespread adoption in healthcare IoT applications. These open issues are identified as follows:

1. Dynamic Adaptation to Body Motion and Interference
  - Unresolved Research Problem: Existing power control either responds reactively (with latency) or requires high computational overhead, failing to achieve proactive, ultra-low-latency adaptation to WBAN’s highly dynamic channels (e.g., human body communication (HBC) channel with significant posture-dependent path loss fluctuations, 60 GHz mm Wave channel vulnerable to environmental noise [26]) and inter-WBAN interference/malicious jamming [27].
  - Future Direction: Develop low-overhead proactive adaptation algorithms integrating channel prediction (e.g., leveraging kinematics/bio-signals [27] to predict posture-induced channel changes) and anti-interference mechanisms, compatible with 60 GHz mm Wave and HBC channel characteristics [26].
2. Energy-QoS Trade-offs
  - Unresolved Research Problem: Heterogeneous medical traffic (strict-latency emergency data vs. delay-tolerant routine data [26]) lacks differentiated power control strategies; the conflict between energy saving (for wearable/implantable nodes with non-replaceable batteries [27]) and transmission reliability (critical for medical diagnosis) remains unbalanced.
  - Future Direction: Design QoS-aware hierarchical power control frameworks, where high-priority data adopts reliability-first power allocation and low-priority data uses energy-optimized strategies, referencing the differentiated QoS design in WBAN applications [26].
3. Multi-Objective Optimization in Dense Deployments
  - Unresolved Research Problem: In practical scenarios such as hospitals, gyms, or rehabilitation centers, multiple wireless body area networks (WBANs) often operate simultaneously within close proximity, leading to severe cross-network interference and resource contention. This makes it challenging to simultaneously optimize energy consumption, latency, throughput, and fairness [28]. Although various mechanisms—including game-theoretic power control, mean-field multi-agent reinforcement learning, and hybrid edge-assisted frameworks—have partially addressed coexistence challenges, these solutions often rely on simplified interference models or require explicit coordination mechanisms, making them ill-suited for truly dense and heterogeneous deployment environments. Few practical coordination schemes simultaneously achieve sensor priority allocation and real-time transmission power adjustment in truly dense, heterogeneous deployments [26].
  - Future Direction: Propose distributed multi-objective optimization algorithms (inspired by cluster head selection-routing coupling optimization [26]) to balance interference suppression, load balancing, and energy efficiency in hybrid-topology dense WBANs [28]. Future research should focus on developing lightweight, standards-compliant coexistence protocols that enable autonomous interference prediction, dynamic channel hopping, and real-time scheduling without centralized control. Integrating real-time spectrum sensing with adaptive power control, alongside developing fairness metrics for multiple WBANs, is crucial for ensuring reliable performance in congested real-world scenarios.
4. Scalability Constraints in Large-Scale Deployments
  - Unresolved Research Problem: Current research literature lacks in-depth analysis of the scalability of power control mechanisms (including signaling overhead, collection time, and coordination costs) in large-scale dense deployment scenarios such as hospitals and gyms. Although many strategies demonstrate considerable energy-saving potential in isolated or small-scale simulations, their practical performance in scenarios with tens to hundreds of coexisting wireless body area networks remains inadequately validated. Regarding signaling overhead, distributed and cooperative approaches (e.g., game theory, multi-agent reinforcement learning) often require frequent exchange of control information (channel status, interference levels, policy updates). In dense networks, such overhead may consume substantial bandwidth and energy, offsetting efficiency gains from adaptive power control. Regarding convergence time, iterative algorithms (e.g., distributed optimization, non-cooperative games) may face prolonged convergence as network scale increases. In highly dynamic body-surface channel environments, slow convergence leads to delayed power decisions, increased packet loss, and energy waste. Regarding coordination costs, centralized or edge-assisted frameworks introduce latency and dependency on coordinating nodes, while fully decentralized approaches may yield suboptimal performance or system instability without carefully designed local rules. In mobile large-scale scenarios, the trade-offs between optimality, coordination costs, and robustness remain poorly understood.
  - Future Direction: Research should prioritize scalable designs, developing lightweight signaling protocols, fast-converging distributed algorithms, and robust coordination frameworks to ensure efficiency and reliability in truly large-scale real-world multi-wireless body area networks.
5. Energy Harvesting Stability
  - Unresolved Research Problem: Energy harvesting (EH) in WBANs commonly utilizes ambient sources such as radio frequency (RF) radiation, kinetic motion, and thermal gradients from the human body or environment. Each type presents distinct characteristics: RF [25] harvesting offers continuous but low-power density [28]; kinetic harvesters generate intermittent bursts correlated with movement; thermal harvesters provide steady but modest voltage differences. Key integration challenges include miniaturization, efficient power conversion, and seamless coupling with sensor electronics without compromising wearability. Moreover, the predictability of harvested energy remains problematic, as it depends on highly variable factors such as user activity patterns, posture changes, and environmental conditions, making stable power budgeting difficult.
  - Future Direction: Future energy harvesting-aware power control must not only adapt to real-time energy supply conditions but also integrate predictive models tailored to the dynamic characteristics of different energy sources to reliably ensure quality of service (QoS). Develop hybrid energy harvesting prediction models (combining human motion patterns [28] and environmental factors) and adaptive power allocation strategies that dynamically adjust transmission parameters based on real-time harvested energy.
6. Algorithm Complexity and Resource Constraints
  - Unresolved Research Problem: The deployment of power control algorithms is fundamentally constrained by the computational and energy limits of prevalent WBAN sensor platforms, such as TelosB (TI MSP430 microcontroller, 10 KB RAM) and MICAz (Atmel ATmega128L, 4 KB RAM). These nodes offer limited processing speed, small memory, and minimal idle power, restricting the feasibility of complex optimizations or online learning. For instance, while convex optimization or lightweight PID control can often run in real-time, deeper neural networks or multi-agent reinforcement learning [27] typically exceed available resources, which are unavailable for resource-constrained WBAN nodes (e.g., implantable devices); lightweight alternatives with comparable performance are lacking, and nodes need to reserve resources for security authentication [28].
  - Future Direction: Future research must prioritize the advancement of hardware-aware algorithm design—developing strategies whose memory footprint, cycle consumption, and energy expenditure align with the capabilities of mainstream embedded platforms, thereby enabling advanced power control in resource-constrained real-world WBAN deployments. Design ultra-lightweight power control models (e.g., tinyML-based distillation models) referencing the lightweight design principles of WBAN authentication schemes [28], and optimize algorithm complexity to fit resource-limited nodes.
7. Security and Privacy Preservation
  - Unresolved Research Problem: Power control-related information (channel state information (CSI), power adjustment strategies [28]) may leak sensitive health data; there is no mature mechanism to integrate privacy protection (e.g., differential privacy) with power control, and the linkage between dynamic power adjustment and attack detection/defense [29] is missing.
  - Future Direction: Develop privacy-preserving power control frameworks based on federated learning [29] (to avoid raw data sharing) and integrate lightweight anomaly detection [28] to suppress malicious interference, ensuring data security without degrading energy efficiency.

To tackle these challenges, the following concrete future research directions are proposed:

1. Develop ultra-lightweight AI models (e.g., tinyML or federated knowledge distillation) specifically tailored for on-device power control in resource-limited WBAN nodes, referencing the lightweight design principles of WBAN authentication schemes [28] to strike a balance between algorithm complexity and real-time performance.
2. Explore cross-layer integration of power control with emerging 6G technologies (e.g., intelligent reflecting surfaces (IRS) and cell-free massive MIMO [26]) to enhance the reliability of dynamic body-area channels, which are highly susceptible to posture changes and tissue attenuation.
3. Investigate privacy-preserving power control frameworks by leveraging differential privacy, homomorphic encryption, or secure multi-party computation, building on federated learning-based privacy protection mechanisms for WBANs [29] to prevent sensitive information leakage from channel states and power adjustment strategies.
4. Design advanced hybrid energy management systems that integrate multiple harvesting sources (e.g., piezoelectric, thermoelectric, RF) [27] with adaptive power allocation under guaranteed QoS constraints, thereby improving the stability and sustainability of energy-harvesting WBANs.
5. Conduct extensive real-world trials in diverse clinical and daily life scenarios (e.g., hospital wards, rehabilitation centers, sports training) [28] to validate simulation-based findings, bridge the gap between theoretical research and practical deployment, and provide empirical support for large-scale WBAN applications.

## 5. Conclusions

Wireless Body Area Networks (WBANs) offer vast potential for medical monitoring, sports tracking, and environmental sensing in the healthcare Internet of Things (IoT). However, limited battery life, body motion interference, and complex channels hinder deployment. Power control mechanisms are vital for enhancing energy efficiency, network lifetime, and data reliability in dynamic settings.

This paper reviews strategies including feedback control, convex optimization, reinforcement learning (RL), deep RL (DRL), hybrid frameworks, and federated learning, achieving average energy savings of 20–30% (peak up to 50%) while maintaining QoS requirements. Convex optimization balances energy-QoS; RL/DRL adapts to multi-node dynamics; hybrids like WDO-MEC and DDPG-offloading boost throughput and curb interference in dense areas.

Despite these advances, challenges remain in algorithmic complexity, energy-QoS trade-offs, and health data privacy. Future research should prioritize lightweight, AI-driven power control solutions—such as advanced deep reinforcement learning, federated learning, and edge-assisted hybrid frameworks—to achieve truly scalable, secure, and sustainable WBAN deployments in real-world healthcare IoT systems. Integrating emerging paradigms like cell-free architectures and privacy-enhancing techniques (e.g., differential privacy) will further pave the way for next-generation body area networks.

## Data Availability

No new data were created or analyzed in this study. Data sharing is not applicable to this article as it is a review paper summarizing existing literature on power control mechanisms in wireless body area networks.

## Abbreviations

The following abbreviations are used in this manuscript:

WBAN	Wireless Body Area Network
IoT	Internet of Things
QoS	Quality of Service
MDP	Markov Decision Process
PSO	Particle Swarm Optimization
RL	Reinforcement Learning
DRL	Deep Reinforcement Learning
MEC	Mobile Edge Computing
WDO	Wind-Driven Optimization
DDPG	Deep Deterministic Policy Gradient
PCTO	Power Control and Task Offloading
SINR	Signal-to-Interference-plus-Noise Ratio
TEPC	Throughput-Equalization Power Control
LLM	Large Language Model
BER	Bit Error Rate
ECG	Electrocardiogram
MAC	Medium Access Control
PID	Proportional-Integral-Derivative
LQI	Link Quality Indicator
RSSI	Received Signal Strength Indicator
HMM	Hidden Markov Model
MF-MARL	Mean-Field Multi-Agent Reinforcement Learning
AoI	Age of Information
EH	Energy Harvesting
PDR	Packet Delivery Ratio

## References

[1] Kwak K.S., Ullah S., Ullah N. An Overview of IEEE 802.15.6 Standard. Proceedings of the 2010 3rd International Symposium on Applied Sciences in Biomedical and Communication Technologies (ISABEL 2010).

[2] Movassaghi S., Abolhasan M., Lipman J., Smith D., Jamalipour A. (2014). Wireless Body Area Networks: A Survey. IEEE Commun. Surv. Tutor..

[3] Fernandes D., Ferreira A.G., Abrishambaf R., Mendes J., Cabral J. (2017). Survey and Taxonomy of Transmissions Power Control Mechanisms for Wireless Body Area Networks. IEEE Commun. Surv. Tutor..

[4] Yang Y., Smith D.B., Rajasegaran J., Seneviratne S. (2021). Power Control for Body Area Networks: Accurate Channel Prediction by Lightweight Deep Learning. IEEE Internet Things J..

[5] Gupta B.K., Ghosal S., Ganesan S., Sudhakar T., Mishra A., Pandey N. Energy Harvesting Techniques for Self-Powered Body-Worn Sensors in Wireless Body Area Networks. Proceedings of the 2025 International Conference on Automation and Computation (AUTOCOM).

[6] (2012). IEEE Standard for Local and Metropolitan Area Networks—Part 15.6: Wireless Body Area Networks.

[7] Wang Z., Aggarwal V., Wang X. (2014). Power Allocation for Energy Harvesting Transmitter With Causal Information. IEEE Trans. Commun..

[8] Amjad O., Bedeer E., Ikki S. (2019). Energy-Efficiency Maximization of Self-Sustained Wireless Body Area Sensor Networks. IEEE Sensors Lett..

[9] Rajawat A.S., Goyal S.B., Bedi P., Verma C., Safirescu C.O., Mihaltan T.C. (2022). Sensors Energy Optimization for Renewable Energy-Based WBANs on Sporadic Elder Movements. Sensors.

[10] Hakami V., Dehghan M. (2016). Distributed Power Control for Delay Optimization in Energy Harvesting Cooperative Relay Networks. IEEE Trans. Veh. Technol..

[11] Wang C., Guo K., Hu X. The QoS and Energy Consumption Efficiency Trade-off Model Based on Utility Function in WBAN. Proceedings of the 2021 5th International Conference on Electronic Information Technology and Computer Engineering.

[12] Masadeh A., Wang Z., Kamal A.E. Reinforcement Learning Exploration Algorithms for Energy Harvesting Communications Systems. Proceedings of the 2018 IEEE International Conference on Communications (ICC 2018).

[13] Nasreen M.A., Ravindran S. An Overview of Q-learning based Energy Efficient Power Allocation in WBAN (Q-EEPA). Proceedings of the 2022 2nd International Conference on Innovative Sustainable Computational Technologies (CISCT).

[14] Sharma M.K., Zappone A., Assaad M., Debbah M., Vassilaras S. (2019). Distributed Power Control for Large Energy Harvesting Networks: A Multi-Agent Deep Reinforcement Learning Approach. IEEE Trans. Cogn. Commun. Netw..

[15] Kim B.-S., Shah B., Kim K.-I. (2022). Adaptive Scheduling and Power Control for Multi-Objective Optimization in IEEE 802.15.6 Based Personalized Wireless Body Area Networks. IEEE Trans. Mob. Comput..

[16] Xu F., Yang H.-C., Alouini M.-S. (2022). Energy Consumption Minimization for Data Collection From Wirelessly-Powered IoT Sensors: Session-Specific Optimal Design With DRL. IEEE Sensors J..

[17] Su H., Li Z., Liu X., Lin S. Wind Driven Optimization-Based Power Control Mechanism for Edge-Enabled Body Area Networks. Proceedings of the 2024 Fifteenth International Conference on Ubiquitous and Future Networks (ICUFN).

[18] Li S., Yang H.C., Xu F., Hu H., Hu F. (2024). Energy-Efficient Relay Transmission for WBAN: Energy Consumption Minimizing Design with Hybrid Supervised/Reinforcement Learning. IEEE Internet Things.

[19] Liao Y., Zhou H., Leng C., Su Z., Qin T. (2025). Power Control and Task Offloading Strategies for High-Density Wireless Body Area Networks Based on Deep Reinforcement Learning. Comput. Netw..

[20] Bao B.Q., Anh B.T., Yen V.T.H., Hiep P.T., Le H.-N. (2024). Joint Throughput Equalization Power Control and Cell-Free Model for Enhancing Performance of WBANs. Wirel. Pers. Commun..

[21] Moin A., Thielens A., Araujo A., Sangiovanni-Vincentelli A., Rabaey J.M. (2020). Adaptive Body Area Networks Using Kinematics and Biosignals. IEEE J. Biomed. Health Inform..

[22] Chen D.R. (2024). Integrating IoT in WBANs: An Energy-Efficient and QoS-Aware Approach for Rapid Model-Driven Transmission Power Control and Link Adaptation. Internet Things.

[23] Pant D., Lohani S., Wason M. (2025). A Lightweight, AI-Enhanced Intrusion Detection System for Wireless Body Area Networks Using Unsupervised Anomaly Detection. Int. J. Glob. Innov. Solutions.

[24] Benabderrahmane F., Kerkouche E., Bouchemal N. (2026). Risk-Aware Privacy-Preserving Federated Learning for Remote Patient Monitoring: A Multi-Layer Adaptive Security Framework. Appl. Sci..

[25] Mohammadi R., Shirmohammadi Z. (2025). Optimizing Energy Harvesting in Wireless Body Area Networks: A Deep Reinforcement Learning Approach to Dynamic Sampling. Alex. Eng. J..

[26] Cornet B., Fang H., Ngo H., Boyer E.W., Wang H. (2022). An Overview of Wireless Body Area Networks for Mobile Health Applications. IEEE Netw..

[27] Wankhade M.P., Ganage D.G., Chincholkar Y. (2025). Effective Cluster Head and Routing Scheme Estimation Using Mixed Attention-Based Drift Enabled Federated Deep Reinforcement Learning in Wireless Sensor Networks. Concurr. Comput. Pr. Exp..

[28] Subramani J., Maria A., Rajasekaran A.S., Al-Turjman F. (2021). Lightweight Privacy and Confidentiality Preserving Anonymous Authentication Scheme for WBANs. IEEE Trans. Ind. Inform..

[29] Preethichandra D.M.G., Piyathilaka L., Izhar U., Samarasinghe R., De Silva L.C. (2023). Wireless Body Area Networks and Their Applications-A Review. IEEE Access.

